# The Role of Cytidine Deaminases on Innate Immune Responses against Human Viral Infections

**DOI:** 10.1155/2013/683095

**Published:** 2013-06-25

**Authors:** Valdimara C. Vieira, Marcelo A. Soares

**Affiliations:** ^1^Programa de Oncovirologia, Instituto Nacional de Câncer, Rua André Cavalcanti, No. 37–4 Andar, Bairro de Fátima, 20231-050 Rio de Janeiro, RJ, Brazil; ^2^Departamento de Genética, Universidade Federal do Rio de Janeiro, 21949-570 Rio de Janeiro, RJ, Brazil

## Abstract

The APOBEC family of proteins comprises deaminase enzymes that edit DNA and/or RNA sequences. The APOBEC3 subgroup plays an important role on the innate immune system, acting on host defense against exogenous viruses and endogenous retroelements. The role of APOBEC3 proteins in the inhibition of viral infection was firstly described for HIV-1. However, in the past few years many studies have also shown evidence of APOBEC3 action on other viruses associated with human diseases, including HTLV, HCV, HBV, HPV, HSV-1, and EBV. APOBEC3 inhibits these viruses through a series of editing-dependent and independent mechanisms. Many viruses have evolved mechanisms to counteract APOBEC effects, and strategies that enhance APOBEC3 activity constitute a new approach for antiviral drug development. On the other hand, novel evidence that editing by APOBEC3 constitutes a source for viral genetic diversification and evolution has emerged. Furthermore, a possible role in cancer development has been shown for these host enzymes. Therefore, understanding the role of deaminases on the immune response against infectious agents, as well as their role in human disease, has become pivotal. This review summarizes the state-of-the-art knowledge of the impact of APOBEC enzymes on human viruses of distinct families and harboring disparate replication strategies.

## 1. Introduction

The human immune system is constantly challenged by invading pathogens, against which it acts by eliminating them or reducing their impact once infection is established. Current and emerging viruses constitute an important fraction of these pathogens that are able to develop short to life-long persistent infections, to some of which no protective vaccines are yet available. In this regard, a better understanding of the mechanisms by which innate and adaptive immunity restricts viral infections and/or modulate viral pathogenesis is urged.

The innate immune system constitutes the first line of defense against viruses, initiating an antiviral response. Viruses are recognized by this system primarily through detection of their nucleic acids, either their packaged genome or viral replication intermediates within the infected cell [1]. Toll-like receptors are good examples of the former viral sensing mechanisms, while the latter are represented by RIG-I-like or DAI and AIM2 receptors [2, 3]. These types of recognition induce the transcription of proinflammatory cytokines and type I interferons (IFNs). These, on their hand, activate the expression of hundreds of IFN-stimulated genes (ISGs) which will engage in counteracting virus replication and spread [4].

Among the ISGs, the genes encoding the family of apolipoprotein B mRNA-editing catalytic polypeptide (APOBEC) cytidine deaminases have been largely studied in the last years. Mounting evidence suggest that these enzymes are key players in restricting infections by different viruses. In this review, we summarize the state-of-the-art knowledge of the impact of APOBEC enzymes on human viruses of distinct families and harboring disparate replication strategies.

## 2. The APOBEC Family of Deaminases

**Figure 1:**
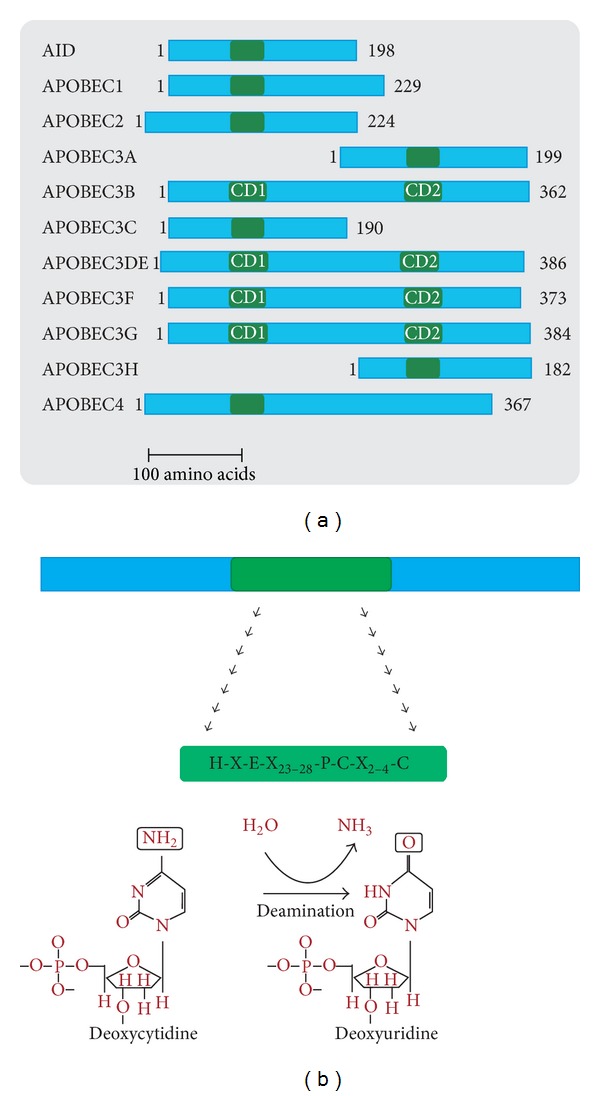
Structure and function of human APOBEC proteins. (a) Schematic representation of APOBEC proteins. The zinc-binding motifs, represented by the catalytic domains (CD) and present in single or double copies, are depicted in green. In the proteins that harbor two CD copies, the N- and C-terminal domains are named CD1 and CD2, respectively. APOBEC proteins are drawn to scale, and the total number of amino acids is shown to the right of each version. The scale bar represents the length of 100 amino acids. (b) The conserved amino acid sequence of the zinc-binding motif is shown; the hydrolytic deamination reaction mediated by these enzymes is shown at the bottom of the figure.

The APOBEC family of proteins comprises a group of cytidine deaminases that are able to edit DNA and/or RNA sequences. Although it belongs to a larger superfamily of deaminases, APOBECs are restricted to vertebrates [5]. In humans, the family comprises eleven members with distinct functions: activation-induced deaminase (AID) and APOBEC1, whose genes are located in chromosome 12; APOBEC2, whose gene is in chromosome 6; seven *APOBEC3* genes, located in chromosome 22; and APOBEC4, whose gene is located in chromosome 1 [6–10]. The members of this family are distinguished by the presence of one or two catalytic domains containing a zinc-binding deaminase motif, characterized by the conserved amino acid sequences H-X-E-X_(23–28)_-P-C-X_(2–4)_-C (X is any amino acid; Figure 1) [9]. The deamination mediated by these enzymes involves the hydrolytic removal of the amino group at the C4 position of a cytidine (C) or deoxycytidine (dC), generating a uridine (U) or deoxyuridine (dU) (Figure 1) [11]. 

APOBEC1 (A1), the first member of the family to be described, is an RNA-editing enzyme [12] but also presents the ability to edit DNA in bacterial assays [13]. It is primarily expressed in the gastrointestinal compartment and catalyzes the posttranscriptional editing of the apolipoprotein B (*apoB*) mRNA. A1 is a nucleocytoplasmic protein [14], and the *apoB* mRNA editing occurs in the nucleus in the presence of the APOBEC-1 complementation factor (ACF) [15, 16]. A1 edits this mRNA at a single base, resulting in the formation of a premature stop codon and leading to the synthesis of a truncated protein (ApoB48) [12]. As a consequence, the human gut produces two forms of ApoB, a longer (ApoB100) and a shorter (ApoB48), involved in the transport of endogenously produced cholesterol and triglycerides and the absorption and transport of exogenous dietary lipids, respectively [17]. Recently, additional mRNA targets of APOBEC1 have been described; the interaction occurs at AU-rich segments of their 3′ untranslated regions. A1 may thus play a role regulating the stability of such specific mRNAs [18, 19]. 

AID, which deaminates single stranded DNA [20, 21], is predominantly cytoplasmic and shuttles between the nucleus and the cytoplasm [22–25]. It is expressed in germinal center B cells and is essential for the events of class switch recombination and somatic hypermutation during the process of antibody diversification [26–28]. AID has also been reported as involved in DNA demethylation [29–31].

APOBEC2 is expressed in heart and skeletal muscles [8], and although its precise physiologic role is not established, its expression appears to be essential to muscle development [32].

The APOBEC3 group comprises seven proteins in humans: APOBEC3A, APOBEC3B, APOBEC3C, APOBEC3DE, APOBEC3F, APOBEC3G, and APOBEC3H. A3A, A3C, and A3H present one copy of the zinc-binding domain, while the remaining harbor two copies; the N- and C-terminal domains are named CD1 and CD2, respectively (Figure 1). In those three enzymes, only CD2 is catalytically active [13, 33, 34]. A3 enzymes are capable of editing single-stranded DNA and recognize specific target sequences. A3G and A3F, for example, edit C's preferentially at CC and TC dinucleotide contexts (GG and AG in the complementary DNA strand), respectively [35–38]. 

A3 enzymes play an important role on the innate immune system, acting on host defense against exogenous viruses and endogenous retroelements [39–42]. Viral restriction occurs mainly by their DNA editing mechanism, but A3s also display editing-independent phenotypes [38, 43–45], as will be discussed later. They are further possibly implicated into a specific pathway of exogenous DNA clearance in human cells [46]. More recently, lines of mounting evidence have also shown that A3 enzymes insert mutations in human nuclear and mitochondrial DNA, suggesting roles in DNA catabolism [47]. On the other hand, this phenomenon may represent a possible source of mutations towards the development of cancer [47, 48]. 

A3 enzymes localize to the cell cytoplasm and/or nucleus, enabling the protection of both compartments through restriction of nuclear (such as the human papillomavirus, the herpes simplex virus, and non-LTR retrotransposons) or cytoplasmic (like the hepatitis B virus, retroviruses, and LTR retrotransposons) replicating elements. A3D, A3F, and A3G are known to be cytoplasmic [40, 49]; A3B localizes to the nucleus [50], while A3A, A3C, and A3H are found both in the nucleus and in the cytoplasm [40, 51]. Noteworthy, different A3H haplotypes present distinct localizations; the protein encoded by haplotype I is mainly nuclear, while the one encoded by haplotype II is predominantly cytoplasmic [51]. With respect to A3A, it has been recently reported that its endogenous version in primary CD14^+^ monocytes and in the monocytic cell line THP-1 localizes to the cytoplasm, contrasting with the broad nucleocytoplasmic distribution observed upon A3A transfection, an observation likely explained by artificial overexpression of the enzyme [52]. 

In addition to presenting distinct subcellular localization, some APOBEC3 proteins also display an intracellular mode of regulation by localization into specific subcellular structures. It is known that A3G is present in two distinct molecular forms within the cell: a form of low molecular mass (LMM) and another in ribonucleoproteic complexes of high molecular mass (HMM) [53–55]. The HMM complex is enzymatically inactive and can be converted into LMM complexes, enzymatically active, through RNase digestion [53]. Besides A3G, other APOBECs like A3C, A3F, and A3H also show the ability of assembling into HMM complexes [56–58]. 

It has been shown that the switch between HMM and LMM can be stimulated by different cytokines [59, 60], and the predominant form varies among different cell types or distinct cell type subsets. The presence of LMM A3G has been related to a reduced susceptibility to HIV-1 infection, as is suggested as postentry restriction factor for this virus [53, 60, 61]. For example, unstimulated peripheral blood CD4^+^ T-cell lymphocytes and monocytes, which are nonpermissive to HIV-1 infection, present LMM A3G. However, when CD4^+^ T-cell lymphocytes are activated or the monocytes stimulated to differentiate into macrophages, they shift their A3G profile to HMM [53]. Noteworthy, the knockdown of A3G in unstimulated CD4^+^ T cells does not turn them permissive to infection, suggesting that the presence of LMM A3G in the cells is not the unique determinant for their resistance to HIV-1 [62, 63]. Moreover, LMM A3G is preferentially packaged into HIV-1 particles [56, 64–66]. Finally, HMM A3G is also able to interact with and sequester *Alu* RNA elements, inhibiting their transposition and evidencing the role of different A3G molecular forms in the restriction of retroelements [67].

A3G and A3F are also able to accumulate in processing bodies (P-bodies) and stress granules, where they interact with RNAs and several proteins that regulate their metabolism [68–71]. However, the functional consequences of the occurrence of A3 proteins in those structures are not yet clear [72].

APOBEC4 is expressed in testicles, and its function is still unknown [10]. Like A2, A4 does not present mutagenic activity in bacterial or yeast assays [73].

APOBEC proteins are found throughout vertebrates, with AID and APOBEC2 being ancestral members of the family and APOBEC1 and APOBEC3 being more recent, while the origins of APOBEC4 are not clear [74–77]. The APOBEC3 enzymes are exclusively found in mammals [5, 78], and their gene copy number is species-specific. While mice have only a single *APOBEC3* gene, pigs have two, sheep and cattle have three, cats have four, horses have six, and primates have at least seven *APOBEC3* genes [5, 9, 79, 80].

The evolutionary history of the *APOBEC3* genes involves expansion, divergence, selection and extinction of specific A3 copies [80]. It is believed that the genome of the mammalian ancestor encoded for at least one ancestral *APOBEC3* gene and that this gene family expanded in the different lineages as a response to changes in viral, retroviral, and retrotransposon pressure [78, 79]. Interestingly, the rapid expansion of the *APOBEC3* locus in primates is correlated with a marked reduction in retrotransposon activity, suggesting an important role in the host genome defense against retroelements [81, 82].

There is evidence that APOBEC3 proteins are able to restrict non-LTR and LTR retrotransposons, including both long interspersed nuclear elements (LINEs) and short interspersed nuclear elements (SINEs) [40, 57, 83–87]. While for some murine LTR retrotransposons, like IAP e MusD, DNA deamination was observed as part of the restriction mechanism [39, 85], the exact mechanism and the retrotransposition step targeted by APOBEC3 is unknown for non-LTR elements (reviewed in [82]).

It is interesting to note that AID and APOBEC1 from multiple species have been shown to possess activity against retroelements [86, 88–90] and exogenous viruses [89, 91–93], suggesting that these proteins may also have a role in innate immunity of some vertebrates [82, 89, 92].

## 3. Role of APOBEC Enzymes on Different Human Viral Infections

### 3.1. Human Immunodeficiency Virus (HIV)

The human immunodeficiency virus (HIV) is a member of the Retroviridae family, and belongs to the *Lentivirus* genus, which characterizes viruses of slow symptomatology. As a retrovirus, HIV harbors a genome consisting of two single-stranded RNA molecules of positive polarity that undergoes a reverse transcription step (through a complementary DNA-cDNA-molecule) carried out by its encoded reverse transcriptase (reviewed in [111]). The cDNA is then integrated into the host cell genome, from where the viral genes are transcribed by the host RNA polymerase II. In addition to the essential genes, HIV also encodes several accessory and regulatory proteins that enhance virus replication and burden, being the viral infectivity factor (Vif) among them (reviewed in [111]). 

**Figure 2:**
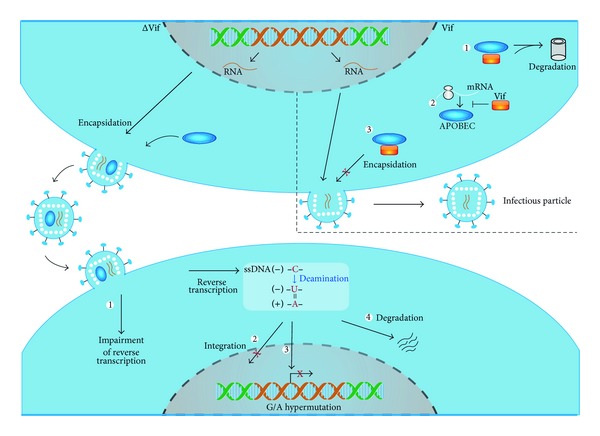
Mechanism of action of APOBEC3 (A3) and Vif in the HIV-1 life cycle. At the top panel, a virus-producing cell in the absence (left) or in the presence (right) of a functional Vif protein is shown. In the presence of Vif (orange rectangle), A3 (blue ellipse) is primarily targeted to proteasomal degradation (1); Vif can also block A3 mRNA translation (2) and prevent A3 packaging into the virion in a degradation-independent manner (3). In the absence of Vif, A3 molecules are packaged into incoming virus particles. After a new infection (bottom panel), A3 exerts its antiviral activity in multiple ways. A3 can interfere with reverse transcription in a deamination-independent manner (1). A3 can also interfere with proviral integration through the formation of abnormal viral DNA ends (2). In the hypermutation process, A3 mainly deaminates dC residues in the negative strand of the complementary viral DNA, originating dU, that serves as template for the incorporation of dA in the positive strand. If able to integrate, hypermutated proviruses are normally largely defective (3). Alternatively, viral DNA containing multiple dU can also be degraded before integration (4).

The hypermutation mediated by APOBEC enzymes in HIV type 1 (HIV-1) is well described (Figure 2). It is known that in HIV-1-infected cells, in the absence of a functional Vif, A3G molecules are incorporated into incoming virus particles. This packaging is mediated by the interaction of A3G with the nucleocapsid (NC) domain of the Gag protein [112–116] and occurs in an RNA-dependent manner [114, 115, 117–120]. After a new infection, the editing process occurs during viral reverse transcription. A3G deaminates dC residues in the negative strand of the complementary DNA (cDNA), originating dU. These nucleotides serve as templates for the incorporation of dA in the positive strand and are evidenced as G-to-A changes in the proviral DNA. The frequency of edited dC's in the viral genome can exceed 10% of the sites [38, 121]. The excessive number of changes results in loss of genetic information and production of largely defective virions in the subsequent replication cycle.

 A reduction of viral reverse transcription products is also observed in the presence of A3G. It has been hypothesized that the presence of dU's in the retroviral DNA could be recognized as anomalous, leading to its degradation even before its integration into the host cell genome (Figure 2). This would occur by removal of the uracil residues by uracil-DNA glycosylases (UDGs), followed by apurinic/apyrimidinic (AP) endonuclease-mediated degradation [122, 123]. Noteworthy, it has been shown that APOBEC-mediated restriction occurs even in the absence of UDGs such as UNG2 and SMUG1 [124, 125], leaving the requirement of retroviral DNA degradation for virus restriction as an open question. 

 In addition to restricting viral infection through hypermutation, A3G also exert editing-independent mechanisms of restriction. These involve the interference with reverse transcription and with proviral integration by disturbing tRNA primer annealing and removal, DNA synthesis initiation and elongation and strand transfer reaction [43–45, 126–135]. 

 Further to the direct mechanisms of viral inhibition, A3G appears to play a pivotal role in the activation of the host immune system. It has been shown that the generated pool of defective viruses encoding truncated or misfolded proteins represents an important source of viral antigens, associated with a strong activation of HIV-1-specific CD8+ cytotoxic T-cells [136]. APOBEC3 may also enhance the recognition of HIV-infected cells by natural killer cells through the activation of the DNA-damage repair response by viruses harboring uridines in their genomes [137]. 

 HIV-1, like the majority of lentiviruses, counteracts the restriction mediated by the A3 enzymes through expression of the Vif protein [138, 139]. The main mechanism of action assigned to Vif is the induction of A3G protein degradation via proteasome. Vif simultaneously binds to A3 and to an E3 ubiquitin-ligase complex, leading to polyubiquitination of A3G and its consequent degradation [138–141]. Vif is also able to prevent A3G packaging into the virion in a degradation-independent manner and to interfere with A3G mRNA translation, thus reducing the intracellular levels of the protein (Figure 2) [142–147].

 APOBEC3F (A3F) has also been associated with HIV restriction in a consistent manner. However, A3G seems to have a more important role in viral restriction of cells targeted by HIV, whereas the role of A3F seems to be dispensable for virus restriction in these cells [148, 149]. A3A, A3B, A3C, A3DE, and some A3H haplotypes have also been implicated in restriction [148, 150–157], although controversial results have been observed (reviewed in [158]).

 HIV hypermutated proviral DNA sequences have been reported in several *in vivo* studies. Yet some reports have shown a correlation between hypermutation and a favorable clinical outcome, this relationship is not consensual. In a population level analysis of HIV-1 subtype B near full-length proviral sequences, hypermutation levels were associated with reduced pretreatment viremia [101]. In agreement with this, higher hypermutation levels were observed in patients with low HIV viral loads (<10,000 copies/mL for at least 3 years) in another study [159]. The presence of hypermutation was also correlated with higher CD4^+^ T-cell counts [160]. More recently, Kourteva et al. [161] found more A3G-hypermutated sites in proviral sequences derived from HIV long-term nonprogressors (LNTP) compared to noncontrollers (NC). On the other hand, some studies could not find such associations, either in adults or in children [162–164].

A more consistent association has been observed between the APOBEC3 mRNA levels and clinical outcomes. Several studies showed a positive correlation of A3 levels with CD4^+^ T-cell counts and a negative correlation with HIV-1 viral load [159, 161, 165, 166] or viral set point [167]. However, two studies could not find an association between A3G levels and CD4^+^ T-cell counts or viral load [104, 168]. APOBEC3G expression has also been inversely correlated with provirus burden [161] and positively correlated with the level of G-to-A changes [159, 161, 162]. A3G has also been found significantly increased in HIV-exposed uninfected (EU) compared to healthy controls [159, 169] or infected individuals [169]. The level of expression in EU significantly decreased after one year from HIV diagnosis and subsequent treatment of their partners. This suggests that, in these individuals, exposure to HIV can trigger APOBEC3G expression in the absence of infection and that the expression decreases with cessation of exposure [159]. Interestingly, the higher expression of APOBEC3G in EU was seen not only in PBMCs but also in cervical tissues, and may be important for the susceptibility to sexually transmitted HIV infection [169].

Higher levels of APOBEC3G mRNA were observed in LTNPs when compared to HIV-uninfected subjects and progressors [165]. Accordingly, higher levels of A3G and A3F were found in LTNP when compared to noncontrollers [161], and higher levels of A3G and A3B were also found in slow progressing patients (SP) when compared to AIDS patients [166]. However, in a group of perinatally HIV-infected children, no correlation was observed between A3G/A3F expression and disease progression [164]. Paradoxically, A3G levels were higher in HIV-negative when compared with HIV positive individuals [104, 166, 168], including matched pre- and postinfection samples from the same subjects. This may suggest that APOBEC3G transcription is rapidly downregulated upon HIV-1 infection [104]. In view of these confounding evidence, additional work is necessary to robustly define the role of A3 expression in the control of HIV infection and disease progression. 

Due to the potential antiviral role of the A3 enzymes, therapeutic interventions have been idealized to enhance their action and lead to viral inhibition through hypermutation [170–174]. In this sense, molecules have been identified that are able to interact with A3G and counteract Vif-mediated degradation [171] or to induce Vif degradation in the presence of A3G [172]. On the other hand, it is possible that the editing mediated by the APOBEC enzymes, upon sublethal conditions (which do not drive virus extinction), acts in the diversification of the viral genome, providing source for the selection and evolution of highly fit variants. This can favor, for example, the acquisition of immune system escape mutations or drug resistance mutations [75, 175–182]. In this regard, novel therapeutic strategies based on complete A3G inhibition, eliminating this additional source of virus diversification, have also been proposed [174, 183, 184]. 

### 3.2. Human T-Cell Lymphotropic Virus (HTLV)

HTLV is a complex retrovirus belonging to the *Deltaretrovirus* genus. In most cases infection by HTLV-1 is asymptomatic; however, up to 5% of the infected subjects develop adult T-cell leukemia (ATL) and another 1-2% develop HTLV-1-associated myelopathy/tropical spastic paraparesis (HAM/TSP) [185, 186].

Like HIV-1, HTLV targets mainly CD4^+^ T lymphocytes [187], and therefore, it would be exposed to several A3 enzymes that are expressed in this cell type [188]. HTLV does not encode any product with Vif-like, A3 antagonist function, and it is apparently incapable of inducing A3 degradation in cell culture [34, 189]. Unexpectedly, however, hyperedited HTLV sequences appear to be rare. Yet HTLV hypermutated sequences with estimated frequencies between 0.1 and 5% have been observed *in vitro*, no instance of hypermutation was reported in PBMC from HTLV-infected patients [34]. Moreover, a retrospective analysis of previously published HTLV sequences has identified a single hypermutated sequence recovered from an HTLV-1 infection of an animal model [190].

Although rare *in vivo*, HTLV hypermutated sequences were recovered from cell lines derived from ATL and HAM/TSP patients. The main context of the observed changes was GG, followed by GA and GC, suggesting the involvement of A3G and also of other A3 such as A3A and A3B in HTLV hypermutation [189]. Despite the fact that A3G is able to edit the viral genome [191, 192], some studies have shown that HTLV-1 is resistant or poorly susceptible to this enzyme [34, 189, 192, 193], in agreement with the low frequency of editing observed *in vivo*. 

Two possible reasons for the low frequency of hypermutated HTLV sequences *in vivo*, when compared to HIV hypermutation, have been discussed. One of the possibilities resides in the differences of replicative strategies of these two viruses. After primary infection, HTLV presents a low level of productive replication, and the proviral genome is mainly replicated through oligoclonal expansion of infected cells, a fact that contrasts with the high rate of *de novo* cell infection seen during chronic HIV disease [185, 194–196]. Therefore, it has been suggested that the infrequent replication via reverse transcription seen in HTLV infection represents a reduction of opportunities for APOBEC3-mediated edition to occur. Moreover, a direct resistance mechanism to A3G has been described in HTLV-1. Through a *cis*-acting exclusion mechanism, elements in the C-terminal region of the HTLV-1 nucleocapsid inhibit A3G packaging to the particle, resulting in reduced efficiency of its packaging in HTLV-1 particles when compared to HIV Δvif virus-like particles [197].

Recently, an analysis of proviral genomes of 60 ATL patients and asymptomatic carriers showed that G-to-A changes are the most frequent nucleotide substitutions. These changes occurred preferentially in the target context of A3G and were involved in the generation of multiple missense substitutions. It was then suggested that HTLV-1-infected cells can take advantage of A3G activity to escape the host immune system by abrogating the expression of viral proteins [191].

### 3.3. Hepatitis B Virus (HBV)

 HBV belongs to the Hepadnaviridae family and presents a circular, partially double stranded DNA genome [198]. It replicates through reverse transcription of an intermediate pregenomic RNA. It is estimated that two billion people have been infected and more than 240 million have chronic liver infections worldwide [199]. HBV infection can cause acute and chronic liver disease, including cirrhosis and hepatocellular carcinoma [198].

 A number of studies have shown that the HBV genome is susceptible to human APOBEC enzymes. In addition to A1 and AID, all A3 enzymes except for A3DE were able to edit the viral genome *in vitro* [200–206] with editing levels estimated between 10^−2^ and 10^−5^ [205]. Viral genome editing occurs preferentially in the negative DNA strand resulting in G-to-A changes in the positive strand; C-to-T changes have also been observed in the positive strand, evidencing editing of both viral DNA strands [202].

 In addition to the identification of HBV sequences extensively edited by APOBEC enzymes, several studies have shown evidence of restriction to virus replication *in vitro*, by both editing-dependent and -independent mechanisms [201, 207, 208]. In this sense, a strong inhibitory effect of A3G has been described [201, 207, 208], and a reduction of approximately 30-fold in the levels of viral DNA in the presence of A3G expression has been shown [204]. Turelli et al. [207] showed that A3G leads to a reduction of viral DNA and also of core-associated RNA; this effect was sustained when a catalytically-inactive A3G mutant was used. Among the possible deamination-independent mechanisms of action are the inhibition of pregenomic RNA packaging and the interference of reverse transcription [201, 207, 208]. Additional unrelated factors may account for the suppression of HBV replication in hepatocytes, including the inhibition of HBV transcription through the interaction of A3B with the heterogeneous nuclear ribonucleoprotein K (hnRNP-K), a positive regulator of HBV expression [209]. 

In the healthy human liver, low to moderate APOBEC expression levels are observed [9, 188, 210]. However, some of these enzymes, particularly A3G, have their expression significantly increased in primary hepatocytes and in hepatoma cell lines in response to stimulation by interferon alpha and gamma [210–212]. In agreement with those data, low hypermutation levels (from 10^−4^ to 0.6%, depending on the method used) have been described in patients with acute and chronic HBV infection [202, 213, 214]. This has led to the hypothesis that hypermutation is intrinsic to the natural response to HBV infection and that it may contribute to the noncytolytic clearance of HBV [202, 207, 210]. However, there are no current *in vitro* evidence supporting a major role of A3 on the IFN-mediated HBV inactivation in the liver [215, 216].

In contrast to the moderate levels of APOBEC expression in the healthy liver, overexpression of these enzymes is observed in cirrhotic tissues, likely resultant from the high production of cytokines associated with the chronic inflammatory response against the infection. Consequent to this increase in APOBEC expression, high levels of HBV hypermutation have been observed, reaching 35% of the sequences in some cases [206].

A potential role of APOBEC3 has been suggested on the oncogenesis of the hepatocellular carcinoma (HCC). It has been shown that some APOBEC3 enzymes are able to generate truncation mutants of the HBx viral protein, leading to a selective advantage to preneoplastic and neoplastic hepatocytes [217]. Moreover, A3B was found to be overexpressed in HCC tissues. In HepG2 cells, A3B overexpression promoted their growth and led to an upregulation of the heat shock transcription factor-1 (HSF-1), which was also found to be upregulated in HCC [217]. HSF-1 is a regulator of the heat-shock response and is known to facilitate malignant transformation, cancer cell survival, and proliferation in model systems. Recently, it has been shown to coordinate a transcriptional program in malignancy, which differs from that induced by thermal stress. This program activates cancer-specific genes that support oncogenic events and was found to be active in breast, colon, and lung tumors [180].

### 3.4. Hepatitis C Virus (HCV)

HCV belongs to the Flaviviridae family and presents a positive sense, single-stranded RNA genome [218]. HCV is a causative agent of acute and chronic liver diseases. Chronic HCV infection, along with chronic HBV infection and its associated liver cirrhosis, constitutes major risk factors for HCC development [219]. It has been recently shown that A3G is able to inhibit HCV replication *in vitro*. However, viral hypermutated sequences have not been found, suggesting a role of deaminase-independent mechanisms of viral inhibition. Considering that A3G targets ssDNA, such lack of hypermutation is expected for HCV, which presents exclusively RNA as genomic material during all phases of its replication. Interestingly, it has also been shown that the presence of exogenous HIV-1 Vif led to an intracellular decrease of A3G and consequently to an increase of HCV replication, suggesting the involvement of Vif in the HIV-1/HCV coinfection [220].

As previously mentioned, the APOBEC enzymes are expressed at moderate levels in the normal liver. However, HCV infection is also associated to an increase in expression of those enzymes. In patients chronically infected by HCV, a significant increase in A3G expression was seen in hepatocytes and in lymphocytes [221]. Overexpression of APOBEC has also been observed in HCV/HBV coinfection [206]. 

APOBEC3 also appears to play an important role on treatment with exogenous interferon alpha (IFN*α*) *in vivo*. Jiménez-Sousa et al. [222] analyzed the profile of gene expression in HCV chronically infected patients after 12 weeks of treatment with IFN*α*/ribavirin (RBV), and A3A was among the IFN-induced genes that was upregulated in early responders but not in nonresponders. In another study, a significant increase in the expression of A3G/3F was observed in CD4 T-cell lymphocytes of HIV/HCV coinfected patients during treatment with pegIFN/RBV. In that study, APOBEC3 induction was correlated with the levels of HIV hypermutation [223].

In addition to the HCV restriction phenotype during the natural course of infection and during treatment, APOBEC3 enzymes have also been suggested as a putative target in anti-HCV drug development. Treatment of HCV-infected Huh7.5 cells with two stabilizing components of APOBEC3G, which increased its intracellular levels, inhibited HCV replication [220].

### 3.5. Human Papillomavirus (HPV)

HPV belongs to the Papillomaviridae family and presents a circular, double-stranded DNA genome. Infection by HPV is a necessary condition for the development of cervical cancer, but the evolution to invasive carcinoma only occurs in a fraction of the infected women [224].

Vartanian et al. [225] showed that both strands of HPV DNA are susceptible to APOBEC editing. In that study, nine HPV16-positive precancerous cervical and six HPV1a-positive plantar wart samples were analyzed for the presence of hypermutation. Of the samples, two HPV-16 and one HPV-1a presented edited sequences. *In vitro* A3A, A3C and A3H were shown to be able to hyperedit HPV DNA. The preferred *in vitro* dinucleotide context for these three A3 enzymes correlated with the editing contexts observed *in vivo*, suggesting that APOBEC3A, APOBEC3C, and APOBEC3H may be involved in edition of HPV *in vivo*. 

### 3.6. Human Herpesviruses (HHV)

 Human APOBEC enzymes have also been shown to restrict DNA genome viruses belonging to the Herpesviridae family [226, 227]. Herpesviruses are enveloped viruses harboring a double-stranded DNA genome. They are associated with a range of different diseases and are able to establish latent infection and persist in the infected host for life [228–231].

Herpes simplex virus type 1 (HSV-1) can cause from mild infections of mucous membranes, including herpes labialis and genital infections, to life threatening infections, such as HSV encephalitis [228, 229]. Suspène et al. [226] have identified the presence of HSV hypermutated genomes in four out of eight oral lesions. Overexpression of A3C *in vitro* led to a fourfold reduction in viral titers and to a 10-fold reduction in viral infectivity. Moreover, it has been shown that not only A3C but also AID, A3A, and A3G are able to edit the HSV-1 genome *in vitro*, although the last three had no significant impact on virus replication. 

In addition to APOBEC3, APOBEC1 has also been shown to restrict HSV-1 replication *in vitro *in a significant fashion, in both deamination-dependent and -independent ways. Upregulation of A1 has been observed in rat brain tissues upon HSV-1 infection, suggesting that A1 induction during encephalitis can promote restriction to HSV-1 infection [227]. 

Epstein-Barr virus (EBV) can cause mucocutaneous manifestations in infectious mononucleosis and is also associated with other benign and malignant conditions, including plasmablastic lymphoma, oral hairy leukoplakia, posttransplant lymphoproliferative disorders, Burkitt's lymphoma, and Hodgkin's lymphoma [231]. In order to know whether EBV genomes were also susceptible to A3 editing, Suspène et al. [226] analyzed EBV from transformed peripheral blood mononuclear cell lines, which carry EBV in a latent form. Edited EBV DNA was found in four out of five EBV cell lines studied. A3C was found to be the most abundantly expressed A3 in these cell lines.

## 4. Polymorphisms in APOBEC3 Genes and Susceptibility to Viral Infections

Polymorphisms in the genes encoding APOBEC proteins have been associated with the modulation in the course of some human viral infections. A deletion of approximately 29.5 kb located between exon 5 of *APOBEC3A* and exon 8 of *APOBEC3B*, which results in the complete removal of the *APOBEC3B* coding region, has been found in different human populations. Its frequency varies among ethnic groups, being more prevalent in East Asians, Amerindians, and Oceanic populations (36.9%, 57.7%, and 92.9%, resp.) but rare among Africans and Europeans (0.9% and 6%, resp.) [232]. This polymorphism has been associated with increased risk for persistent HBV infection and for the development of HBV-associated hepatocellular carcinoma. Due to the A3B ability of restricting HBV, it has been suggested that this gene deletion can result in reduced viral clearance, culminating with persistent infection [97]. In other studies, the homozygosity status for the deletion was associated with mild liver fibrosis, but not with a chronic carrier status [95], and with faster progression of liver disease [98]. Homozygous individuals for this deletion have also been reported with an increased risk for HIV acquisition, for a higher viral set point and for progression to AIDS [94], yet no effect was found on susceptibility to HIV infection and AIDS in Japanese or Indian populations [96].

Several polymorphisms in the *APOBEC3G* gene have also been described. In a study with 3,073 HIV patients from 6 different cohorts, a variant at *APOBEC3G *exon 4, H186R, was frequently found in African Americans (37%) but rare in European American (<3%) and in Europeans (5%). This polymorphism was associated with CD4^+^ T-cell decline and with accelerated progression to AIDS-defining conditions in African Americans [99]. In another study with South African infected women, H186R was associated with higher HIV viral loads, and an extragenic mutation (rs35228531) was associated with decreased CD4^+^ T-cell levels [104]. In a study with HIV-1-infected children from Pediatric AIDS Clinical Trials Group (PACTG) protocols P152 and P300, the H186R and F119F variants were associated with altered HIV-1-related disease progression and central nervous system impairment [108]. However, no correlation was found between H186R and disease progression in a French cohort [100]. This polymorphism has not been found in Indians [103]. Another *APOBEC3G* gene polymorphism, C40693T, was associated with an increased risk of HIV infection in a cohort of 122 Caucasian individuals highly exposed to HIV-1 [102]. Finally, the *APOBEC3G* SNP-571 (rs5757463) was associated with lower CD4^+^ T-cell counts in homo- and heterozygosis in a group of HIV-1-infected individuals naive to drug therapy from Brazil [106].

*APOBEC3H* gene is also polymorphic, with seven haplotypes identified in human populations so far (named from I to VII). Of them, only three (II, V, and VII) seem to originate a stable A3H protein, and have a higher anti-HIV-1 activity [156, 157, 233]. *APOBEC3H* haplotype II has also been reported to potently restrict HTLV-1 [189]. Some of these haplotypes have a variable frequency. Haplotype 2 is present in high frequency in African populations [233]; haplotype V is more frequently detected in African-Americans, Caribbeans, and Chinese, while haplotype VII was rarer and found only in European Caucasians [157].

A comprehensive list of studies that described the association of particular *APOBEC* gene variants with viral infections of disease outcomes can be seen in Table 1. Despite several of these so-called candidate gene analysis studies identified definite associations, particularly in the context of HIV and HBV infections, they were not confirmed in genome-wide association studies (GWAS). The latter studies point mainly to a significant role of human leukocyte antigen (HLA) alleles [234–239], suggesting that the impact of *APOBEC* variations on viral diseases is less robust and only moderate [234].

## 5. Concluding Remarks

 Innate immunity mechanisms are the first line against invading viruses and are promising targets for preventing viral infections. Some infections are life-long once established, for example, by HIV and HSV-1, and therefore innate immunity is pivotal to avoiding the unfavorable consequences of infection of the host. The APOBEC family of cytidine deaminases plays an important role within innate immunity by deteriorating the genetic information of many viruses through hypermutation-dependent and -independent mechanisms. Polymorphisms in *APOBEC* genes that render differences in their expression and enzymatic activity also affect viral infection outcomes, and yet currently these effects appear modest and are not evidenced by GWAS. Viruses, on their hand, evolved molecular mechanisms to counteract APOBEC effects. Diminished APOBEC activity and sublethal levels of hypermutation may favor virus evolution, by generating viable variants carrying immune escape or drug resistance mutations. Conversely, the unfavorable consequences of APOBEC upregulation, particularly its recently described carcinogenic and genotoxic potential, are important caveats that will require further assessment and will pose a challenge to strategies aiming at increasing APOBEC expression to counteract viral infections. These issues will certainly warrant continuing investigation on the role and effects of cytidine deamination in infectious diseases and cancer.

## Figures and Tables

**Table 1:** Studies that investigated the association of *APOBEC* gene variants and the course of human viral infections.

A3 family member	Series description	Main findings	Reference
A3B	4,216 individuals from five HIV-1 natural history cohorts based in the United States of America	Homozygous deletion associated with increased risk for HIV-1 infection (*P* = 0.24), progression to AIDS (*P* = 0.03), and viral set point (*P* = 0.04)	[94]

A3B	724 HBV carriers and 469 healthy control subjects	*APOBEC3*B deletion homozygosity was associated with mild liver fibrosis (*P* = 0.0019) No significant association between deletion and chronic HBV infection	[95]

A3B	361 Japanese subjects: 95 HIV-1-infected patients (48 nonprogressors and 47 slow progressors) and 266 controls 453 Indian subjects: 251 HIV-1-infected patients and 202 controls	No evidence of association between the *APOBEC3B* deletion and susceptibility to HIV infection and AIDS	[96]

A3B	1,124 individuals with HCC, 510 individuals with persistent HBV infection, and 826 healthy controls. All subjects were of Han Chinese ethnicity	Higher frequency of the *APOBEC3B* deletion allele in persistent HBV carriers (*P* = 0.0015) and HCC patients (*P* = 1.28 × 10^−11^) compared to controls Presence of at least one deletion allele was associated with an increased risk for persistent HBV infection (*P* = 0.0272) and HCC development (*P* = 1.28 × 10^−11^)	[97]

A3B	179 HBV chronic carriers and 216 healthy control subjects from the Moroccan population	No significant difference in the frequency of deleted *APOBEC3B* alleles between patients with chronic hepatitis B and control subjects Subjects carrying the Del/Del genotype displayed a trend for increased susceptibility to HBV infection compared to the wild type genotype (*P* = 0.07) Carriers of the *APOBEC3B* deletion had significantly lower viral loads than patients with the wild type genotype (*P* = 0.0023)	[98]

A3G	3,073 participants enrolled in six HIV/AIDS prospective cohorts: 1,481 European Americans, 949 African Americans from five US-based cohorts, and 643 patients enrolled in the Swiss HIV cohort	For African Americans, the variant allele 186R was strongly associated with a decline of CD4^+^ T cells (*P* = 0.009)	[99]

A3G	773 white French individuals: 327 HIV-1^+^ (245 slow progressors; 82 rapid progressors) and 446 healthy control subjects of similar ethnic origin	29 polymorphisms with allele frequencies >1% were identified No significant associations were found between the polymorphisms or haplotypes and disease progression	[100]

A3G	136 adult HIV-infected patients from the Western Australian HIV cohort	22 single nucleotide polymorphisms were identified No significant association of these* APOBEC3G* genetic variants and the presence of HIV-1 hypermutation was found (although an intronic allele 6892C was marginally associated with HIV-1 hypermutation)	[101]

A3G	122 Caucasian individuals exposed to HIV enrolled in prospective cohort studies in Montreal	The C40693T variant was significantly associated with an increased risk of infection (*P* = 0.03)	[102]

A3G	560 North Indians: 50 HIV-1 exposed seronegative individuals, 190 HIV-1^+^ patients, and 320 healthy controls	No H186R polymorphism of *APOBEC3G * was found among North Indians	[103]

A3G	250 South African women at high risk for HIV-1 subtype C infection	The H186R mutation and a 3′ extragenic mutation (rs35228531) were associated with high HIV viral loads (*P* = 0.0097 and *P* < 0.0001) and decreased CD4^+^ T-cell counts (*P* = 0.0081 and *P* < 0.0001)	[104]

A3G	534 children perinatally exposed to HIV-1 (109 exposed uninfected and 425 HIV-1-infected), from a pediatric cohort of white-Hispanic ethnicity from Argentina	HIV-1 perinatal transmission and progression to AIDS were not affected by *APOBEC3G* H186R or *APOBEC3G* C40693T *APOBEC3G* C40693T was correlated with substitutions in Vif motifs involved in the interaction with APOBEC3G (*P* = 0.004)	[105]

A3G	400 HIV-1-infected individuals naive to drug therapy from the Brazilian population	Seven *loci* were analyzed: SNP −571 (rs5757463); −199 (rs34550797); −90 (rs5750743); 119 (rs5757465); 186 (rs8177832); 197 (rs3736685); 199 (rs2294367) For the SNP −571, heterozygous (C/G) and homozygous (G/G) individuals had lower CD4^+^ T-cell counts compared to homozygous (C/C) individuals (*P* = 0.0076)	[106]

A3G	93 perinatally infected children with white-Hispanic ethnicity, from an Argentinian pediatric cohort	The *APOBEC3G* H186R and *APOBEC3G *C40693T variants were not associated with different levels of HIV-1 editing	[107]

A3G	1,049 HIV-1-infected children from the Pediatric AIDS Clinical Trials Group (PACTG) protocols P152 and P300 (60% non-Hispanic black, 26% Hispanic, 13% non-Hispanic white, and 1% other or unknown race/ethnicity)	*APOBEC3G* H186R homozygous G/G genotype was associated with faster HIV-1 disease progression (*P* = 0.01) and central nervous system (CNS) impairment (*P* = 0.02) *APOBEC3G *F119F-C allele was associated with protection against disease progression and CNS impairment in both additive and dominant models (*P* = 0.002 and *P* = 0.001, resp.) and CNS impairment (*P* = 0.02 and *P* = 0.007, resp.)	[108]

A3G	179 HBV chronic carriers and 216 healthy control subjects from the Moroccan population	No significant difference in the frequencies of *APOBEC3G* H186R genotype between patients with chronic hepatitis B and control subjects	[98]

A3H	70 Italian HIV-exposed seronegative individuals and their HIV-1-infected sexual partners	The *APOBEC3H *haplotype I was found in a higher frequency in the exposed seronegative compared to the HIV^+^ individuals (*P* = 0.0056), suggesting a protection from sexually transmitted HIV-1 infection	[109]

A3H	96 recently HIV-1-infected treatment-naïve adults	68 SNPs were analyzed Homozygous carriers of an *APOBEC3H* risk haplotype (A3Hrh) had lower GA→AA (A3F) sequence editing on proviral HIV-1 *vif *sequence (*P* = 0.01) and lower HIV-1 RNA levels (*P* = 0.015)	[110]
